# PW01-029 – Relationship between apoptotic alterations and inflammation in familial Mediterranean fever

**DOI:** 10.1186/1546-0096-11-S1-A82

**Published:** 2013-11-08

**Authors:** G Mkrtchyan, A Boyajyan

**Affiliations:** 1 Institute of Molecular Biology of NAS RA, Yerevan, Armenia

## Introduction

A number of studies indicated that alterations of apoptosis and its regulation together with upregulated inflammatory response are involved in pathogenesis of familial Mediterranean fever (FMF). However, molecular and cellular mechanisms responsible for abnormalities in apoptosis and their relationship with autoinflammatory responses in FMF are not clear.

## Objectives

In the present study we determined the levels of annexin-A5, ficolin-H, and ficolin-L proteins, in the blood of patients with FMF and healthy controls. Assessment of correlation between the levels of these proteins was also performed. Annexin-A5 is a marker of apoptosis; ficolin-H and ficolin-L are components of the complement cascade. Binding of ficolins to the surface of apoptotic cells may activate the complement lectin pathway, as well as phagocytosis of apoptotic cells. Thus, ficolins may act as both inflammatory mediators and opsonins.

## Methods

Forty four FMF-affected subjects and 50 healthy controls were involved in the study. The enzyme linked immunosorbent assay was used to measure blood serum levels of annexin-A5, ficolin-H, and ficolin-L proteins in patients and controls. Statistical approaches included Mann-Whitney U test and Spearman correlation analysis.

## Results

Significantly increased levels of annexin-A5, ficolin-H, and ficolin-L proteins were detected in FMF-affected subjects as compared to healthy controls (p<0.05). The data obtained provided further evidence on the increased rate of apoptosis in FMF and demonstrated the involvement of the complement lectin pathway in FMF-associated inflammatory reactions. In addition, in case of FMF a positive correlation between the levels of annexin-A5 and ficolin-H, as well as between the levels of annexin-5 and ficolin-L. No correlation between the measured parameters was detected in healthy subjects group. These results indicated that the detected correlation in conditioned by FMF-associated pathologic processes, namely increased rate of apoptosis and upregulated inflammation.

## Conclusion

The obtained results suggest that the pathogenesis of FMF is characterized by increase rate of apoptosis associated with hyperactivation of the complement lectin pathway. Based on the obtained data we also concluded that apoptotic alterations and upregulated inflammation in FMF are interrelated.The obtained results suggest that the pathogenesis of FMF is characterized by increase rate of apoptosis associated with hyperactivation of the complement lectin pathway. Based on the obtained data we also concluded that apoptotic alterations and upregulated inflammation in FMF are interrelated.The obtained results suggest that the pathogenesis of FMF is characterized by increase rate of apoptosis associated with hyperactivation of the complement lectin pathway. Based on the obtained data we also concluded thatapoptotic alterations and upregulated inflammation in FMF are interrelated.

## Disclosure of interest

None declared

